# Psychometric Assessment of the Chinese Version of the Supportive Care Needs Survey Short-Form (SCNS-SF34-C) among Hong Kong and Taiwanese Chinese Colorectal Cancer Patients

**DOI:** 10.1371/journal.pone.0075755

**Published:** 2013-10-11

**Authors:** Wylie Wai Yee Li, Wendy Wing Tak Lam, Shiow-Ching Shun, Yeur-Hur Lai, Wai-Lun Law, Jensen Poon, Richard Fielding

**Affiliations:** 1 Centre for Psycho-Oncology Research and Training, Division of Behavioural Health, School of Public Health, The University of Hong Kong, Hong Kong; 2 Department of Nursing, National Taiwan University, Taipei, Taiwan; 3 Department of Surgery, The University of Hong Kong, Hong Kong; Federal University of Rio de Janeiro, Brazil

## Abstract

**Background:**

Accurate assessment of unmet supportive care needs is essential for optimal cancer patient care. This study used confirmatory factor analysis (CFA) to test the known factor structures of the short form of Supportive Care Need Survey (SCNS-34) in Hong Kong and Taiwan Chinese patients diagnosed with colorectal cancer (CRC).

**Methods:**

360 Hong Kong and 263 Taiwanese Chinese CRC patients completed the Chinese version of SCNS-SF34. Comparative measures (patient satisfaction, anxiety, depression, and symptom distress) tested convergent validity while known group differences were examined to test discriminant validity.

**Results:**

The original 5-factor and recent 4-factor models of the SCNS demonstrated poor data fit using CFA in both Hong Kong and Taiwan samples. Subsequently a modified five-factor model with correlated residuals demonstrated acceptable fit in both samples. Correlations demonstrated convergent and divergent validity and known group differences were observed.

**Conclusions:**

While the five-factor model demonstrated a better fit for data from Chinese colorectal cancer patients, some of the items within its domain overlapped, suggesting item redundancy. The five-factor model showed good psychometric properties in these samples but also suggests conceptualization of unmet supportive care needs are currently inadequate.

## Introduction

Unmet supportive care needs provide specific information about the physical and psycho-social needs of cancer patients [1] and thus can optimise medical service utilization by targeting clinical care to unmet need, such as symptom control and specific rehabilitation challenges [2]. Multiple studies have investigated the supportive care needs of different cancer groups at different time points throughout the cancer journey across different cultures [1]–[8].

Colorectal cancer (CRC) currently the second most prevalent cancer in Hong Kong's population is projected to be the most prevalent cancer within 5 years [9]. As patient numbers increase, so too do associated health care costs. Diagnosis and treatment of CRC not only affects patients physically but also substantially impacts their quality of life [10], [11], psychological wellbeing [12] and body image [13] at considerable financial cost. Because significant individual and cultural differences exist in these impacts [8] then reliably assessing unmet supportive care needs becomes crucial to cost-effective care provision.

The Supportive Care Needs Survey (SCNS) was developed in Australia by Girgis and colleagues [14] for assessing cancer patients' unmet needs. The Short-Form Supportive Care Needs Survey (SCNS-SF34) reportedly has good internal validity and reliability [15]. The SCNS-SF34 had been translated and validated for use in different language and cultural communities, including Chinese [16], German [17], French [18], and Japanese [19]. The German (SCNS-SF34-G) and Japanese (SCNS-SF34-J) produced factor-loading patterns comparable to the original SCNS-SF34 among groups of breast and prostate cancer and breast cancer patients respectively [17], [19]. Subsequently, Schofield et al [20] has reported a similar SCNS-SF34 factor structure among Australian prostate cancer patients using a revised four-point response format. Brédart et al [18] used confirmatory factor analysis (CFA) to uphold a five-factor structure for the SCNS-SF34-Fr in French and Swiss breast cancer patients. However, among Hong Kong breast cancer patients using the Chinese version (SCNS-SF34-C), exploratory factor analysis showed a four-factor structure from 33 items provided a better data fit [16].

Except for the French version, studies have only examined the factorial structure of the SCNS-SF using an exploratory factor analysis in which no model is specified prior to the analysis. The main objective of the present study was to further validate the SCNS-SF by evaluating its factorial structure in a Hong Kong Chinese sample of patients with colorectal cancer (CRC). We compared the fit of the original five-factor model with the fit of the 4-factor model as proposed by Au et al [16]. Second, we examined whether the factorial structure of the SCNS-SF extends to a sample of Taiwanese Chinese CRC patients who share a similar cultural background, but different health care system. Taiwan uses a national health insurance approach while Hong Kong uses a mixture of public and private health services. There is evidence that Hong Kong and Taiwan CRC patients report different health system and information related needs and psychological needs, with Hong Kong patients reporting greater unmet health system and information related needs and Taiwanese patients greater psychological unmet needs [8]. Third, we also tested the convergent validity, discriminant validity, and internal reliability of the SCNS-SF using both Hong Kong and Taiwan samples.

## Methods

### Participants

Local ethics approval was independently obtained for recruitment and consent procedures from National Taiwan University Hospital and Hong Kong University/Hospital Authority HK West Cluster Institutional Review Boards and Ethics committees. All eligible participants gave fully informed written consent regarding study purpose, data confidentiality and rights to refusal and uncontested withdrawal.

#### Hong Kong

Chinese patients diagnosed with colorectal cancer attending a surgical out-patient clinic in Hong Kong, between September 2009–January 2012, were screened by clinicians for eligibility. Consecutive sampling was adopted. Eligibility criteria were Cantonese/Mandarin fluency, either current receiving active treatment or had completed active treatment, willingness and ability to complete the interview and age 18 years or older. Eligible participants completed face-to-face interviews administered by trained research assistants while waiting for follow-up consultation or before primary surgery. Patients lacking Cantonese/Mandarin fluency and those functionally incapable were excluded.

#### Taiwan

Consenting patients were recruited from outpatient oncology and surgical clinics of a leading medical centre in northern Taiwan. Consecutive sampling was adopted. All were ≥18 years old, diagnosed and fully informed about their CRC and either still receiving or had completed active treatment, and able to communicate verbally.

### Core Measure

#### Supportive care needs

The SCNS-SF34 Chinese version was used [21]. This measure comprises five domains: Physical and daily living needs (5 items PDL), Psychological needs (10 items PSY), Patient care and support needs (5 items PCS), Health systems and information needs (11 items HSI) and Sexuality needs (3 items SEX). Participants report the magnitude of each specified need over the past month on a 5-point Likert scale (1 = no need, not applicable; 2 = no need, satisfied; 3 = low need; 4 = moderate need; 5 = high need).

### Comparative measures (Hong Kong Sample)

#### Patient satisfaction with care

The Chinese Patient Satisfaction Questionnaire (ChPSQ-9) measures out-patient clinic users' satisfaction with doctors' and nurses' performance [22]. Patients rate their satisfaction on a 5-point Likert scale ranging from ‘Very satisfied’ to ‘Very dissatisfied”, with higher scores indicating lower patient satisfaction. This instrument has good internal validity and internal reliability in cancer patients (Doctor subscale: Cronbach's α = 0.92–0.94; Nurse subscale: Cronbach's α = 0.86–0.89) [23], [24].

#### Psychological distress

The 14-item Hospital Anxiety and Depression Scale (HADS) [25] comprises two 7-item subscales measuring anxiety (HADS-A) and depression (HADS-D). Summing all 14 items gives a total score (HADS-T). Patients were asked to give a 4-point categorical response referenced over the past week. The Chinese version of HADS has adequate internal reliability (HADS-T: Cronbach's α = 0.81; HADS-A: α = 0.80; HADS-D: α = 0.63) and concurrent validity [26].

#### Symptom distress

The Memorial Symptom Assessment Scale–Short Form (MSAS-SF) [27] is a self-reported instrument assessing distress associated with 28 physical and psychological cancer-related symptoms, and the frequencies of four psychological symptoms during the past 7 days. Five-point Likert response options, ranging from ‘0 = not at all’ to ‘4 = very much’ assess patient's physical and psychological symptom distress. This scale comprises four subscales: Global Distress Index (GDI), Physical Symptom Distress Score (PHYS), Psychological Symptom Distress Score (PSYCH), Number of Symptoms score and Total MSAS. The Chinese version (Ch-MSAS-SF) has good validity and reliability (Total MSAS: Cronbach's α = 0.91; GDI: α = 0.85; PHYS: α = 0.84; PSYCH: α = 0.85) [28].

### Comparative measures (Taiwan sample)

#### Psychological distress

As in the Hong Kong sample, the Chinese version of HADS was also used to assess psychological distress in the Taiwan sample.

#### Symptom distress

The 23-item Modified Symptom Distress Scale (SDS) was used to assess symptom distress. This scale with 23 items was modified from the symptom distress scale [29], [30]. Five-point Likert-type scale ranging from 1 (no distress at all) to 5 (as much distress as possible) assesses symptom distress, with higher scores indicates greater symptom distress. Cronbach's α for the SDS in this study was 0.865.

The above comparative measures were used to assess convergent validity. Socio-demographic and medical data were also obtained from patients and checked against their medical records.

### Procedure

#### Hong Kong

Following informed consent, participants completed a combined questionnaire orally-administered by trained research assistants to minimize respondent literacy problems.

#### Taiwan

Following informed consent, SCNS-SF34 data were collected during follow-up out-patient clinic visits for cancer-related treatment, or one month after completion by trained interviewers.

### Data analysis

To assess the factorial validity of the 5-factor and the 4-factor models of the SCNS identified previously, Confirmatory factor analysis (CFA) was performed using Mplus 5.21 software [31]. CFA was tested using maximum likelihood estimation of the sample covariance matrix. The Chi-squared test, sensitive to sample size [32], was supplemented with the root mean square error of approximation (RMSEA), standardised root mean square residual (SRMR) and comparative fit (CFI) indices [33]. RMSEA, a badness-of-fit index should approach zero for the best fit [33]. RMSEA values <0.06 to <0.08 with 90% confidence interval were adopted [34]. The general cut-off criterion for SRMR was ≤0.08 and CFI was ≥0.90 for acceptance respectively [35].

Cronbach's alpha coefficient and item-to-total correlation were used to assess internal consistency with the minimal acceptable alpha specified at 0.7 [36]. Item internal consistency was reached if the correlations between items within a subscale ≥0.40. Item discriminant validity was supported if the correlations were higher with its own subscale than other subscales [37].

Convergent validity, the extent to which theoretically-related measures are correlated with each other, was evaluated by correlating (using Pearson's correlation analysis) SCNS-SF34-C domains with HADS, MSAS-PHY, MSAS-PSYCH, and ChPSQ-9 in the Hong Kong sample and with the HADS and Modified Symptom Distress scale in the Taiwan sample. We hypothesised that SCNS-SF34-C scores would correlate as follows: SCNS-34 HSI and PCS domains would positively correlate with ChPSQ-9 (poor patient satisfaction) because they both measures support received from the health care system. PSY and PDL domains would positively correlate with HADS, (greater psychological distress), MSAS-PHY, MSAS-PSYCH, Modified Symptom Distress scale (greater physical and psychological symptom distress) because these measures assess the experience of physical and psychological concerns.

#### Discriminant validity

We tested Lehmann, Koch & Mehnart's [17] finding of gender differences in SCNS domain scores. We hypothesized that male patients would express stronger SEX domain supportive care needs while female patients would express stronger PSY and PDL domain supportive care needs. To test Jorgensen's [5] finding of age differences in SCNS domain scores, we hypothesized younger patients would express stronger supportive care needs across all domains. Student's *t*-test was used to examine these hypotheses.

## Results

### Sample characteristics

#### Hong Kong sample

A total of 360/416 Hong Kong Chinese patients were eligible to participate in this study. Their mean age was 65.7 years (SD = 11.1) (Table 1) and 227 (63.1%) were male. Most patients had achieved secondary education level (37.9%), a majority were married or cohabiting (76%) and were retired or unemployed (75%). Most patients were not receiving active treatment at the time of recruitment (95.5%) with 57% awaiting primary surgery.

**Table 1:** Socio-demographic and clinical characteristics.

Characteristics	Hong Kong sample (n = 360)	Taiwan sample (n = 263)
Age - years		
Mean (Standard deviation)	65.7 (11.1)	58.4 (11.2)
Range	27–90	23–82
Gender (%)		
Male	227 (63.1)	150 (57)
Female	133 (36.9)	113 (43)
Education level (%)		
No formal education	66 (18.4)	11 (4.2)
Primary education	115 (32.0)	54 (20.5)
Secondary education	136 (37.9)	89 (33.8)
Tertiary education	42 (11.7)	109 (41.5)
Marital status (%)		
Single	26 (7.2)	28 (10.6)
Married/cohabiting	272 (76.0)	210 (79.8)
Separated/divorced	19 (5.3)	6 (2.2)
Widowed	41 (11.5)	19 (7.2)
Occupation (%)		
Full-time	77 (21.4)	78 (29.7)
Part-time	13 (3.6)	14 (5.3)
No job	269 (75)	171 (65)
Cancer status (%)		
Newly diagnosis	328 (91.1)	237 (90.1)
Recurrent	9 (2.5)	26 (9.9)
Missing	23 (6.4)	
Treatment status (%)		
No active treatment	343 (95.3)	163 (62)
Active treatment	16 (4.4)	100 (38)
Chemotherapy	16 (100)	100 (100)
Targeted therapy	4 (25)	20 (20)
Missing	1 (0.3)	-
Surgery status (%)		
No surgery received	4 (1.1)	10 (3.8)
Awaiting surgery	206 (57.2)	-
Completed surgery	150 (41.7)	253 (96.2)
Had colostomy	51 (34)	29 (11)

#### Taiwan sample

A total of 263/298 Taiwanese Chinese patients were eligible to participate in the study. Of the 263 Taiwanese CRC patients, 150 were male (57%) and 113 were female (43%), with a mean age of 58.4 years (SD: 11.2, range: 23–82) (Table 1). Two-fifths were educated to tertiary level (41%), 80% were married or cohabiting, and 65% retired or unemployed. Most patients (62%) were not receiving active treatment when recruited while 96% had completed primary surgery.

### Missing data

There was no missing SCNS data in the Taiwan sample, whereas only 0.19% of data SCNS were missing in the Hong Kong sample. No significant differences were found between patients for whom all SCNS items were complete and those with missing data in terms of medical and socio-demographic characteristics.

### Confirmatory factor analysis

Both original 5-factor SCNS-SF 34 and 4-factor SCNS-33-C models were tested using the Hong Kong sample, then cross-validated using the Taiwan sample. Table 2 summarized the goodness-of-fit indices of the four models. These indicated that both 5-factor and 4-factor models in both Hong Kong and Taiwan samples failed to meet the minimum fit criterion.

**Table 2:** Confirmatory Factor Analysis (CFA), goodness-of-fit indices of Supportive care needs survey (SCNS-SF).

Model	*χ* ^2^	*df*	*p*-value	CFI	SRMR	RMSEA	90% CI
Hong Kong sample							
SCNS-SF34	1270.794	517	<0.001	0.854	0.062	0.064	0.059, 0.068
SCNS-SF33-C	1389.987	489	<0.001	0.824	0.066	0.072	0.067, 0.076
Modified SCNS-SF34*	979.854	509	<0.001	0.909	0.060	0.052	0.046–0.055
Taiwan sample							
SCNS-SF34	2082.170	517	<0.001	0.776	0.093	0.107	0.102–0.112
SCNS-SF33-C	7293.622	528	<0.001	0.714	0.089	0.123	0.118–0.128
Modified SCNS-SF34**	1216.159	503	<0.001	0.898	0.070	0.073	0.068–0.079

SCNS-SF34: original 34 item 5-factor model; SCNS-SF33-C, Chinese 33 item 4-factors model; *χ* ^2^, chi-square statistics; df, degrees of freedom; CFI, comparative fit index; SRMR, standardised root mean square residual; RMSEA, root mean square error of approximation; CI, confidence interval.

* This modified model allowed correlations between residuals of 9 pairs of items within a same factor, including item 4 and item 5, item 6 and item7, item 7 and item 8, item 6 and item 8, item 12 and item 13, item 12 and item 14, item 9 and item 14, item 27 and item 28, item 23 and item 32.

** This modified model allowed correlations between residuals of 9 pairs of items within a same factor, including item 6 and item 7, item 7 and item 8, item 9 and item 10, item 10 and item 11, item 12 and item 13, item 18 and item 19, item 20 and item 22, item 27 and item 28, item 32 and item 33.

Since the 4-factor model did not demonstrate a better fit than the original 5-factor model, modification indices were used to improve the fit of the original five-factor model [38]. Modification indices suggested allowing correlations between residuals (i.e. measurement errors) of several pairs of items within the same factor domain. In both Hong Kong and Taiwan samples, residuals of 9 pairs of items within a same factor were allowed to correlate (Table 2). It appears that the correlated residual items were due to similar concerns being addressed in the corresponding questions, suggesting the possibility that item redundancy/similarity existed in the respective factor domain(s). For example, both item 4 “work around the home” and item 5 “not being able to do things you used to do” assessed patients' ability to perform their daily tasks; item 7 “feeling down or depressed” and item 8 “feelings of sadness” assessed depressive symptoms. For both Hong Kong and Taiwan samples, most item redundancy (6 out of 9 pairs in Hong Kong sample; 5 out of 9 pairs in Taiwan sample) existed between PSY domain items.

The modified model based on the Hong Kong sample was substantially improved revealing an adequate fit to the data. The fit of the modified model based on the Taiwan sample also improved, but the model only reached a marginally adequate fit to the data. The standardized factor loadings of the 5-factor model for Hong Kong and Taiwan samples are presented in Table 3.

**Table 3:** Confirmatory factor analysis of modified SCNS-SF34 – factor loadings pattern.

SCNS items	Factor loadings	
	Hong Kong Sample	Taiwan Sample
Physical and daily living needs		
1. Pain	0.664	0.560
2. Lack of energy/tiredness	0.783	0.820
3. Feeling unwell a lot of the time	0.753	0.789
4. Work around the home	0.390	0.307
5. Not being able to do the things you used to do	0.542	0.672
Psychological needs		
6. Anxiety	0.634	0.714
7. Feeling down and depressed	0.653	0.743
8. Feeling of sadness	0.647	0.806
9. Fears about the cancer spreading	0.723	0.698
10. Worry that the results of treatment are beyond your control	0.832	0.722
11. Uncertainty about the future	0.799	0.797
12. Learning to feel in control of your situation	0.638	0.702
13. Keeping a positive outlook	0.379	0.726
14. Feelings about death and dying	0.640	0.714
17. Concerns about the worries of those close to you	0.484	0.474
Sexual needs		
15. Changes in sexual feelings	1.00	0.991
16. Changes in your sexual relationships	0.724	0.957
31. To be given information about sexual relationships	0.336	0.628
Patient care and support needs		
18. More choice about which cancer specialists you see	0.472	0.190
19. More choice about which hospital you attend	0.256	0.427
20. Reassurance by medical staff that the way you feel is normal	0.730	0.800
21. Hospital staff attending promptly to your physical needs	0.763	0.970
22. Hospital staff acknowledging, and showing sensitivity to, your feelings and emotional needs	0.795	0.907
Health system and information needs		
23. Being given written information about the important aspects of your care	0.654	0.667
24. Being given information (written, diagrams, drawings) about aspects of managing your illness and side-effects at home	0.556	0.706
25. Being given explanations of those tests for which you would like explanations	0.751	0.875
26. Being adequately informed about the benefits and side-effects of treatments before you choose to have them	0.666	0.869
27. Being informed about your test results as soon as feasible	0.653	0.881
28. Being informed about cancer which is under control or diminishing (that is, in remission)	0.703	0.757
29. Being informed about things you can do to help yourself to get well	0.725	0.289
30. Having access to professional counselling (e.g. psychologist, social worker, counsellor, nurse specialist) if you, your family or friends need it	0.540	0.506
32. Being treated like a person not just another case	0.646	0.535
33. Being treated in a hospital or clinic that is as physically pleasant as possible	0.546	0.659
34. Having one member of hospital staff with whom you can talk to about all aspects of your condition, treatment and follow up	0.681	0.379

### Reliability

The reliability of the 5-factor model of the SCNS-SF is presented in Table 4. The internal consistency for the 5-factor model was good in the Taiwan sample with Cronbach's alpha over the acceptable value of 0.7 for all five domains, For the Hong Kong sample, the internal consistency was good for all domains except the Sexual needs domain, which had a low Cronbach's alpha of 0.53. The mean scores ranged from 2.01 (SEX domain) to 35.07 (HSI domain) in the Hong Kong sample, and ranged from 4.25 (SEX domain) to 27.41 (HSI domain) in the Taiwan sample.

**Table 4:** Reliability and descriptive data of SCNS-SF34 five-factor model.

	Number of items	Mean (0–100)	Standard Deviation (SD)	Median	Lowest score	Highest score	Item-own scale correlation^a^ (% of correlation >0.40)	Item-other scale correlation^a^ (% of scaling success)^b^	Alpha coefficient
Hong Kong sample									
Physical and daily living needs	5	11.32	14.60	5.00	0	95.00	0.37–0.64 (80%)	0.006–0.45 (100%)	.771
Psychological needs	10	10.52	14.24	5.00	0	94.45	0.36–0.76 (90%)	0.002–0.54 (100%)	.885
Sexual needs	3	2.01	6.95	0.00	0	50	0.29–0.56 (66.7%)	0.05–0.21 (100%)	.534
Patient care and support needs	5	19.46	18.05	15.00	0	100	0.24–0.64 (60%)	0.03–0.55 (80%)	.731
Health system and information needs	11	35.07	23.69	29.54	0	100	0.49–0.72 (100%)	0.04–0.54 (100%)	.887
Taiwan sample									
Physical and daily living needs	5	13.63	14.73	10.00	0	75	0.28–0.67 (80%)	0.05–0.56 (80%)	.761
Psychological needs	10	17.84	17.15	12.50	0	100	0.42–0.80 (100%)	0.03–0.55 (90%)	.971
Sexual needs	3	4.25	11.74	0.00	0	100	0.62–0.89 (100%)	0.10–0.17 (100%)	.887
Patient care and support needs	5	19.70	15.40	15.00	0	100	0.51–0.78 (100%)	0.09–0.63 (80%)	.857
Health system and information needs	11	27.41	18.02	22.72	0	100	0.50–0.85 (100%)	0.01–0.63 (72.7%)	.903

% of scaling success reflects proportion of subscale items correlating more with subscale of origin items than with items from other subscales.

a Corrected for overlap.

b Scaling success = percentage of cases where the item-own scale correlations are significantly higher than the item-other scale correlations.

In the Taiwan sample, item internal consistency (item-own scale correlations ≥0.40) was seen for all items within each domain, excepting the PDL domain. The proportion of items meeting the .40 criterion in the PDL needs domain was 80%. In contrast, for the Hong Kong sample only the HSI domain had all items meeting the criterion, with the proportion of items meeting the .40 criterion in the PDL, PSY, SEX, and PCS domains ranging from 60% to 90%.

In the Hong Kong sample, item-other scale correlations showed that 100% of all the items correlate more strongly with their own domain items than with other domains' items, supporting item discriminant validity. Likewise, in the Taiwan sample, most items, ranging from 72.7% to 100%, in each domain correlated significantly more with their own domain than with other domains.

### Convergent validity

To assess scale convergent validity, the five-factor model of the SCNS-SF was correlated with measures of anxiety, depression, and symptom distress in both the Hong Kong and Taiwan samples, and patient satisfaction with clinical staff in the Hong Kong sample only (Table 5). As hypothesized, the HSI and PCS domains demonstrated stronger correlations with PSQ-9 satisfaction scores than with measures of anxiety, depression, and symptom distress, while PSY and PDL domain scores correlated more strongly with measures of anxiety, depression, and symptom distress.

**Table 5:** SCNS-SF34 5-factor model domains and anxiety, depression, symptom distress, patient satisfaction with clinical staffs and optimism, Pearson's correlation.

	Health System and information needs	Psychological need	Physical and daily living	Patient care and support needs	Sexual needs
Hong Kong sample					
Anxiety (HADS A)	.390***	.623***	.556***	.375**	.171**
Depression (HADS D)	.310***	.514***	.616***	.320***	.118*
Physical distress (MSAS Physic)	.315***	.481***	.665***	.336***	.161**
Psychological distress (MSAS Psych)	.369***	.621***	.554***	.366***	.140**
Patient satisfaction, nurses (PSQ9)	.256***	.163**	.103	.259***	.096
Patient satisfaction, doctors (PSQ9)	.314***	.151**	.084	.255***	.097
Taiwan sample					
Anxiety (HADS A)	.415***	.698***	.527***	.423***	.087
Depression (HADS D)	.422***	.545***	.497***	.428***	.151*
Symptom distress (Modified Symptom distress scale)	>414***	.657***	.741***	.390***	.137*

*** *p*-value<0.001,

** *p*-value<0.01,

* *p*-value<0.05.

HADS A: Hospital Anxiety and Depression Anxiety subscale; HADS D: Hospital Anxiety and Depression Depression subscale;

MSAS Physic: Memorial Symptom Assessment scale – physical distress subscale; MSAS Psych: Psychological distress subscale;

PSQ9: Patient satisfaction scale.

### Discriminant validity

We next compared the 5-factor SCNS-SF scores by age and gender using Student's *t*-test to examine known group differences in both Hong Kong and Taiwan samples. Significant differences emerged between patients who were aged under 65 and 65 or above for all domains except PDL domain in both samples. Younger patients reported more unmet HSI (Hong Kong: t = 3.80, *p*<0.001; Taiwan: t = 2.38, p = 0.005), PSY (Hong Kong: t = 2.32, *p* = 0.021; Taiwan: t = 2.69, p = 0.008), PCS (Hong Kong: t = 4.93, p<0.001; Taiwan: t = 2.72, p = 0.007) and SEX domains (Hong Kong: *t* = 4.30, *p*<0.001; Taiwan: t = 2.69, p = 0.008) needs than did older patients. In contrast, except for Hong Kong female patients reporting greater unmet PSY (t = −2.53, p = 0.012) and PCS (t = −2.45, p = 0.015) needs and Hong Kong male patients reported greater unmet SEX needs (t = 2.34, p = 0.020), SCNS domain scores did not differ by gender (Table 6).

**Table 6:** Known group differences by SCNS-SF33-C 3-factor model domains.

	Health System and information needs			Psychological need			Physical and daily living			Patient care and support needs			Sexual needs		
	Mean	SD	*p*-value	Mean	SD	*p*-value	Mean	SD	*p*-value	Mean	SD	*p*-value	Mean	SD	*p*-value
Hong Kong sample															
Age			<.001			.021			NS			<.001			<.001
≤65	40.07	25.24		12.36	13.80		12.34	15.14		24.38	20.20		3.65	9.12	
>65	30.66	21.34		8.89	14.46		10.42	14.10		15.10	14.63		0.57	3.63	
Gender			NS			.012			NS			.015			.020
Male	33.65	24.08		9.07	13.33		10.51	13.57		17.69	16.55		2.61	7.74	
Female	37.50	22.90		12.98	15.40		12.71	16.18		22.48	20.06		1.00	5.23	
Taiwan sample															
Age			.005			.008			NS			.007			.008
≤65	29.02	19.40		19.42	17.81		14.48	14.80		20.94	16.93		5.19	12.87	
>65	23.14	12.88		13.65	14.58		11.39	14.44		16.39	9.69		1.74	7.52	
Gender			NS			NS			NS			NS			NS
Male	26.59	16.97		16.05	15.11		12.33	13.36		19.30	15.28		5.22	13.50	
Female	28.50	19.36		20.22	19.35		15.35	16.29		20.22	15.62		2.95	8.76	

SD, standard deviation.

## Discussion

Previous studies examining factor structures of the SCNS-SF34 and its validity as a measure of unmet needs in different samples used mixed groups comprising cancer patient with various diagnoses [15], [17] while others involved cancer patients only with breast [16], [18], [19] or prostate [20] cancer. The original 5-factor SCNS-SF34 model has tended to prevail throughout.

With the exception of the French version of the SCNS-SF [18], previous studies primarily used exploratory factor analysis to examine the factorial structure of the SCNS-SF. The present study assessed the factorial structure of the Chinese version of the SCNS-SF using confirmatory factor analysis in two different colorectal cancer samples, Hong Kong Chinese and Taiwan Chinese patients. We compared the fit of the original 5-factor model with the fit of the 4-factor model proposed by Au et al [16]. In the current study neither the original 5-factor SCNS-SF34 [15] nor the 4-factor SCNS-SF33-C [16] proved a good fit to these two independent Chinese CRC samples, suggesting that one universal SCNS-34 factor structure appears unlikely. Boyes et al's [15] and Schofield et al's [20] Australian, Lehmann et al's [17] German and Bredart et al's [18] French/Swiss samples comprised primarily Caucasian patients raised in western cultural environments. However, both Okuyama et al [19] and Au et al [16] found five and four factor solutions respectively with Asian breast cancer patients, while Lam et al. [7] found significantly different emphasis in unmet supportive care needs between comparable samples of German Caucasian and Hong Kong Chinese women with breast cancer using an optimized 4-factor SCNS-SF33-C structure [16]. The failure to replicate this factor structure among Hong Kong Chinese colorectal cancer patients despite both groups of patients having the same cultural background suggests other effects, such as permutations of culture, age and gender differences, and possibly, but by not means certainly, cancer type, may strongly influence how people experience supportive care needs, interpret symptoms, construe impacts and source support and how much health professionals as opposed to family are expected to meet these.

Since the 4-factor model did not fit better than the original 5-factor model, the original 5-factor model was chosen for closer examination to improve fittingness. Similar to Bredart et al's French version of the SCNS-SF [18], several items within its domains were correlated, suggesting content redundancy. The redundancy of the content was mostly related to the domain measuring psychological unmet needs. These findings highlight a need for refinement of the existing measure aiming to reduce the redundancy of the content. A 9-item brief version derived from the SCNS-SF was recently developed as a screening tool for assessing unmet needs [39]. The 9-item screening version demonstrated adequate sensitivity and specificity in an Australian sample.

Nevertheless, the modified 5-factor SCNS-SF demonstrated an acceptable fit model in both Hong Kong and Taiwan CRC samples. Also, the Chinese version of the SCNS-SF demonstrated good internal consistency for the five domains. Though the SEX domain demonstrated weak internal consistency in the Hong Kong sample. This is due to the item “to be given information about sexual relationships”; assessing information needs correlated weakly with the other two items which assess changes in sexuality. Item internal consistency was also supported by the fact that most items correlated more strongly with their own domains. Item discriminant validity was supported as almost all items correlated higher with its own domain than with other domains.

The Chinese version of the SCNS-SF also showed good convergent validity in both Hong Kong and Taiwan samples demonstrated by strong positive correlation between psychological unmet needs and psychological distress; strong positive correlation between physical and daily living unmet needs and physical symptom distress. Consistent with previous studies on women with breast cancer from France [18], Hong Kong [16], and Japan [19], physical and daily living unmet needs were also strongly correlated with psychological distress, suggesting cancer patients conflate psychological and physical needs related to symptoms. Similar to Bredart et al's study [18], patient satisfaction correlated more with health system and information needs and care and support needs, supporting the convergent validity of the SCNS-SF. However, the strength of these correlations between patient satisfaction and health system and information needs, and between care and support needs were only moderate, reflecting the differences between the concept of patient satisfaction and the concept of unmet needs. Patient satisfaction reflects patients' expectations of services, but does not address exactly what patients needs are. In contrast, needs assessment offers a direct measure of patients' support preferences identifying unmet needs, enabling us to identify gaps in existing services [40].

Known group comparison demonstrated good discriminant validity. CRC patients younger than 65 years reported stronger unmet needs across all domains, except physical and daily living domain compared to older patients, similar to breast cancer patients [16], [17]. Age is a known predictor of unmet supportive care needs strength [41]. As hypothesized, female patients reported stronger unmet Psy domain needs, whereas males reported stronger unmet SEX needs, consistent with Chorost et al's. [42] finding that, following rectal cancer surgery men reported more sexual dysfunction than did women.

The present study showed the extent of unmet supportive care was primarily related to health system and information aspects of care in both Hong Kong and Taiwanese samples. Unmet supportive care in relation to sexual need was minimal in both samples. This is consistent with previous studies demonstrating that patterns of unmet supportive care needs differ across cultures or health care services among Caucasians, Japanese, and Chinese [8]. It is unlikely that low SEX needs were due to unwillingness to discuss sexuality. Previous studies based on Hong Kong Chinese women with breast cancer also showed similar low unmet needs [2], [7], [16]. Furthermore, previous studies on Chinese women with breast cancer had demonstrated no difference in reporting sexuality between using self-administrated format and using face-to-face interview [43]. It is likely that these differences reflect true variation in the values surrounding sexuality in different cultures [8].

The main strength of the present study is the inclusion of two datasets from samples of similar cultural background but geographically diverse Chinese populations which enables us to test the factorial validity across two samples. On the other hand, this study is limited to the recruitment of Hong Kong and Taiwanese Chinese colorectal patients based on one regional public hospital in Hong Kong and Taiwan respectively. A broader sample frame would have been preferable. Secondly, this cross-sectional study prohibited test-retest reliability assessment.

In summary, the present study found that the modified 5-factor structure for 34 items of the Chinese version of the SCNS-SF best fitted the data for two independently recruited samples of CRC patients of Chinese ethnicity. While the internal reliability and clinical validity of the SCNS-SF is consistently demonstrated across studies, the item redundancy limited the factorial validity of the instrument. Hence, caution should be taken using SCNS-SF to assess supportive care needs in other cultural or cancer-type contexts.
